# Variability and Reproducibility of 3^rd^-generation dual-source dynamic volume perfusion CT Parameters in Comparison to MR-perfusion Parameters in Rectal Cancer

**DOI:** 10.1038/s41598-018-25307-w

**Published:** 2018-05-02

**Authors:** Sonja Sudarski, Thomas Henzler, Teresa Floss, Tanja Gaa, Mathias Meyer, Holger Haubenreisser, Stefan O. Schoenberg, Ulrike I. Attenberger

**Affiliations:** 1 Institute of Clinical Radiology and Nuclear Medicine, University Medical Centre Mannheim, Medical Faculty Mannheim, Heidelberg University, Mannheim, Germany; 2 Computer Assisted Clinical Medicine, Medical Faculty Mannheim, Heidelberg University, Mannheim, Germany

## Abstract

To compare in patients with untreated rectal cancer quantitative perfusion parameters calculated from 3^rd^-generation dual-source dynamic volume perfusion CT (dVPCT) with 3-Tesla-MR-perfusion with regard to data variability and tumour differentiation. In MR-perfusion, plasma flow (PF), plasma volume (PV) and mean transit time (MTT) were assessed in two measurements (M1 and M2) by the same reader. In dVPCT, blood flow (BF), blood volume (BV), MTT and permeability (PERM) were assessed respectively. CT dose values were calculated. 20 patients (60 ± 13 years) were analysed. Intra-individual and intra-reader variability of duplicate MR-perfusion measurements was higher compared to duplicate dVPCT measurements. dVPCT-derived BF, BV and PERM could differentiate between tumour and normal rectal wall (significance level for M1 and M2, respectively, regarding BF: p < 0.0001*/0.0001*; BV: p < 0.0001*/0.0001*; MTT: p = 0.93/0.39; PERM: p < 0.0001*/0.0001*), with MR-perfusion this was true for PF and PV (p-values M1/M2 for PF: p = 0.04*/0.01*; PV: p = 0.002*/0.003*; MTT: p = 0.70/0.27*). Mean effective dose of CT-staging incl. dVPCT was 29 ± 6 mSv (20 ± 5 mSv for dVPCT alone). In conclusion, dVPCT has a lower data variability than MR-perfusion while both dVPCT and MR-perfusion could differentiate tumour tissue from normal rectal wall. With 3^rd^-generation dual-source CT dVPCT could be included in a standard CT-staging without exceeding national dose reference values.

## Introduction

Magnetic resonance imaging (MRI) is the accepted gold standard for local staging of rectal cancer prior to treatment^1–4^. Despite the infiltration of the mesorectal fascia being to date the most important T-stage criterion for treatment decisions in colorectal carcinoma, it is known, that molecular subtypes of rectal cancer occur even within the same clinical T-stages do result in heterogeneity in terms of treatment response and overall survival^5^. Therefore new criteria have to be established to better differentiate these patient groups a-priori to treatment as well as during response assessment^6^. One possibility to further characterize rectal cancer lesions in MR beyond morphologic criteria is functional imaging: Quantitative MR-perfusion values were proven to be reproducible within small variation ranges between readers^7^. Previous research suggested a role for MR-perfusion as an imaging-based biomarker for the improved assessment and prediction of therapeutic response alone^8,9^ or together with other parameters as multi-parametric MRI^10–13^ in the neo-adjuvant setting of locally-advanced rectal cancer.

Computed tomography (CT) perfusion values have shown to be robust and reproducible due to linear signal intensity characteristics of the Hounsfield units scale which is a prerequisite for a technique intended to be used for therapeutic response assessment/prediction. Several studies have investigated the accuracy and the additional value of CT perfusion in therapeutic response assessment/prediction in/of different tumour entities also in rectal cancer^14–19^. The main disadvantage of CT perfusion has always been the amount of radiation dose the patient was exposed to.

In the past few years, with the advent of new dose-saving CT scanner generations featuring e.g. noise-reduction algorithms during post-processing, perfusion analysis by means of CT has become a more practicable alternative to perfusion MRI^20,21^.

To the best of our knowledge, dynamic volume perfusion CT (dVPCT) featuring noise-reduction as well as motion-correction algorithms by means of 3^rd^ generation dual-source CT scanner systems was not yet evaluated in direct comparison to MR-perfusion performed on a 3 Tesla MR scanner system in the same patients with primary and untreated rectal cancer. Our study goals were therefore to prospectively investigate quantitative perfusion parameters derived from 3^rd^ generation dual-source dVPCT with regard to intra-individual variability of measurements, intra-reader variability, accurate differentiation of tumour from normal rectal wall and with regard to the possibility to include the perfusion scan in a CT (re-)staging protocol without exceeding radiation dose recommendations for the CT staging.

## Methods

### Study protocol

This prospective single-centre study was performed according to standards of the Health Insurance Portability and Accountability Act (HIPAA) and the Declaration of Helsinki and in accordance with the guidelines of our institutional ethics committee, the “Medizinische Ethikkommission II of the Heidelberg University at the University Medical Centre Mannheim”, which was the committee having approved the study design and study sequences acquired with MRI and CT in the study participants. All study participants gave written informed consent before inclusion in the study. Patients with clinically indicated MR of the pelvis at a 3 Tesla MR scanner system for local staging and clinically indicated staging CT of the chest and abdomen to screen for distant metastases were prospectively and consecutively enrolled. All patients that were asked to participate in the study had undergone coloscopy/recto-sigmoidoscopy a-priori to MR and CT and had suspected rectal cancer.

In case of consent to participate, they underwent a standard staging protocol for rectal cancer including MR-perfusion sequence as reference standard and within 14 days in the framework of their staging for distant metastases by means of contrast-enhanced CT scan of the chest and abdomen, they underwent the additional study CT perfusion sequence over the region of their tumour including a second i.v. contrast bolus.

Inclusion criteria were age >18, and capability of giving consent.

**Figure 1:**
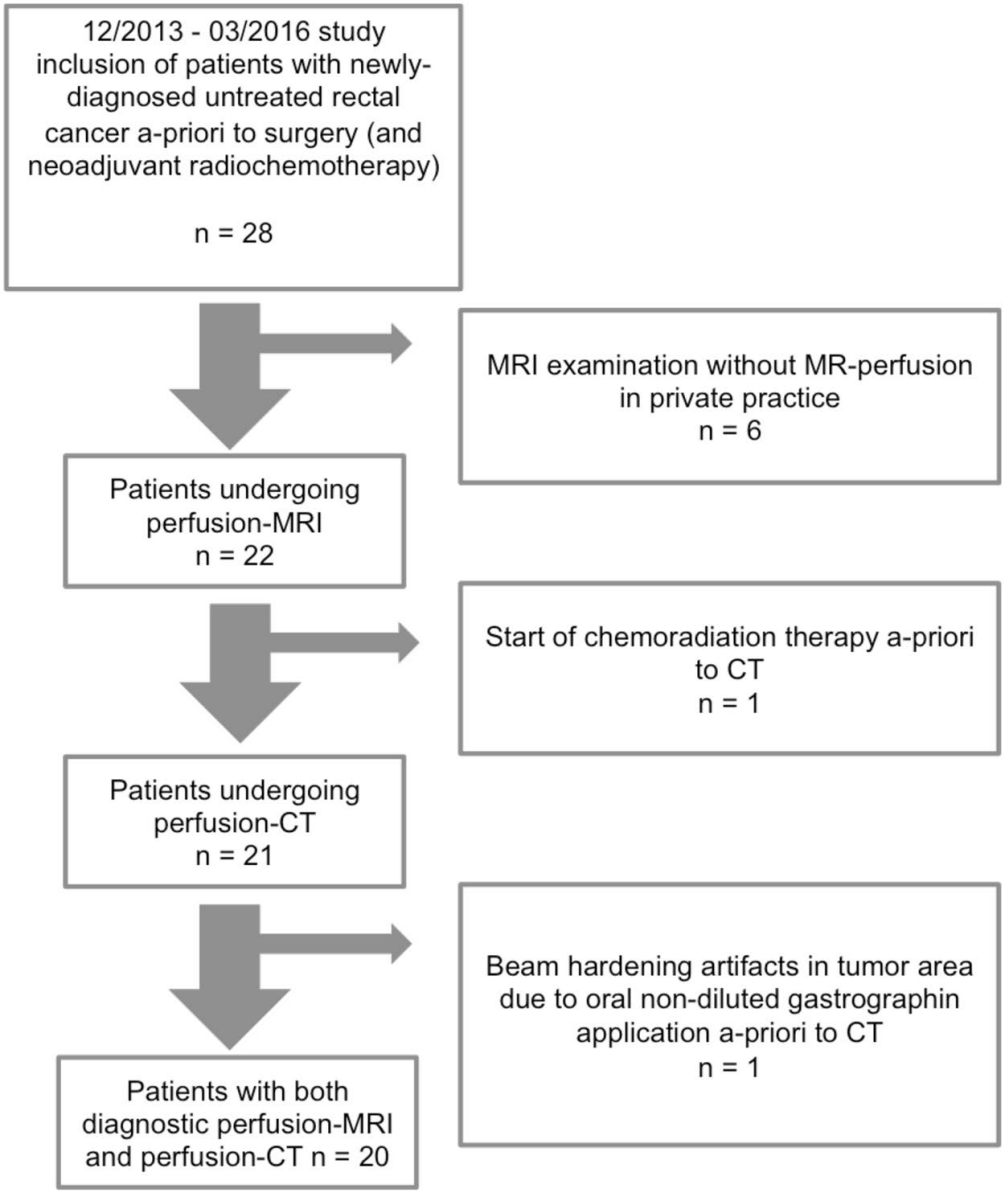
Study patient selection.

A-priori exclusion criteria were CT contrast media allergic reaction history, renal insufficiency, pregnancy. A-posteriori exclusion criteria were >14 days between MR and CT examination, missing or non-diagnostic MR-or CT perfusion sequence e.g. due to insufficient rectal filling because of stenotic tumour or artefacts due to hip implants. 28 consecutive patients were prospectively enrolled. Final analysis could be performed with 20 patients (60 ± 13 years; 14 men, 6 women), see Fig. 1 for study patient selection. According to TNM-classification the investigated tumours were distributed as follows: T1 tumours: 0, T2: 4, T3:15, T4: 1. All patients were revealed to suffer from adenocarcinoma, 3 patients did not undergo surgery. In these 3 patients, histopathological diagnosis was reached by punch biopsy in the course of coloscopy/recto-sigmoidoscopy.

### Data acquisition

MR-Data was acquired on a 3 Tesla scanner system (Magnetom Skyra, Siemens Healthineers, Erlangen, Germany). A 6-element-body-matrix-coil was used and patients got rectal filling with 100 mL of ultrasound gel. They underwent their distinct examination protocol including a 3D TWIST MR-perfusion sequence with the following parameters TR/TE/FA = 3.6 ms/1.44 ms/15°, matrix-size = 192*117, FOV = 259 mm*158 mm, 20 slices. Temporal resolution was 5 s and in total 70 volumes were acquired (Video 1). Gadolinium based contrast agent (Dotarem, Guerbert, France) was injected at a dose of 0.1 mmol/kg after the 10th volume at a flow rate of 1.5 mL/s.

dVPCT was acquired on a 2 × 192 slice dual-source CT system (Somatom Force, Siemens Healthineers, Erlangen, Germany). Patients got rectal filling with 100 mL of ultrasound gel a-priori to CT data acquisition. Depending on patient’s body weight, tube voltage was set to 80, 90 or 100 kVp. A dynamic shuttle-mode perfusion protocol was planned on the a-priori performed CT staging exam of chest and abdomen with the following acquisition protocol in dual-energy technique: tube voltage 90/150 Sn (Sn = use of a tin filter for spectral shaping); tube current time product (ref.) 10/85 mAs; 80 mL (Iodine concentration 400 mg iodine/mL) of i.v. contrast (Imeron, Bracco Imaging, Germany); monophasic chest exam performed during arterial phase; biphasic upper abdomen exam performed during arterial phase and portal-venous phase and monophasic portal-venous phase pelvic exam). The dVPCT scan was performed 15 minutes after the portal-venous phase abdominal scan was completed. 50 mL (Iodine concentration 400 mg iodine/mL) of i.v. contrast (Imeron, Bracco Imaging, Germany) were applied via an 18 G peripheral catheter. Highest temporal resolution during arterial phase was 1.5 s and in total 19 scans were acquired (Video 2). See Table 1 for the detailed CT perfusion protocol.

**Table 1:** Acquisition parameters of the study CT perfusion sequence.

Scan area	Pelvis
Scan mode	VPCT
Scan length	11.4 mm
Scan direction	Adaptive 4D spiral
Tube voltage	80, 90 or 100 kVp
Tube current	220, 150 or 100 mAs, respectively
Dose modulation	—
Rotation time	0.33 s
Cycle protocol	2 × 3 s; 10 × 1.5 s; 3 × 4.5 s; 4 × 9 s
Max. temporal resolution	1.5 s
Total cycle time	64 s
Slice collimation	48 × 1,2 mm
Slice width	1.5 mm
Reconstruction increment	1.0 mm
Reconstruction kernel	Br36
Contrast osmolality	400 mg/mL
Volume	50 mL + 50 mL Saline
Flow rate	5 mL/s
Start delay of scan	5 s after start of contrast injection

kVp = peak kilovoltage; VPCT = volume perfusion CT.

Post-processing was performed on a dedicated work-station a-priori to the perfusion analysis on the dVPCT data stack, consisting of a noise-reduction algorithm as well as a motion correction algorithm (Siemens syngo.via CT Body Perfusion VA30, Siemens Healthineers, Erlangen, Germany).

Dose-length-product of the dVPCT study sequence and of the total CT examination consisting of the staging CT scan and the dVPCT scan were assessed. The total CT examination was designed to adhere to national diagnostic reference values by the German Federal Office for Radiation Protection^22^ for standard a CT chest scan and biphasic abdominal scan valid at the time-point of patient enrolment.

### Image analysis

As morphological gold standard to accurately choose the tumour region and normal rectal wall, high-resolution para-axial T2 TSE MR images were used.

**Figure 2:**
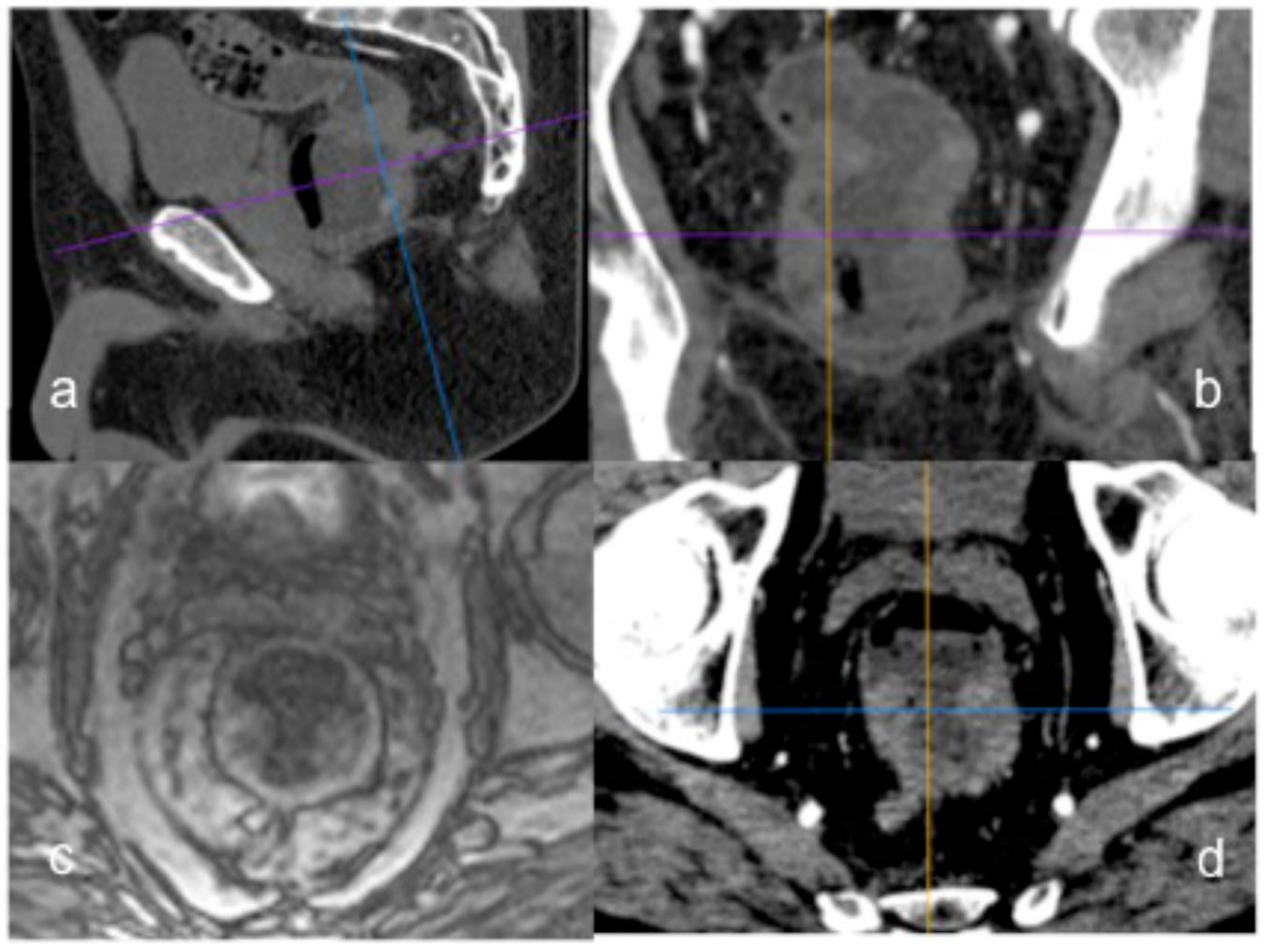
Example of corresponding rectal cancer tumour areas in MR and CT and localization of the investigated tumour area within the pelvis: (**a**) Sagittal view of the rectal carcinoma and localization of the the representative tumour area investigated with perfusion analysis. (**b**) Coronal view of the rectal carcinoma and localization of the representative tumour area investigated with perfusion analysis. (**c**) Paraaxial MR image from the T1 weighted TWIST angiographic stack perpendicular to the rectum lumen, with representative semicircular tumour area at 3 to 9 o’clock suitable for tumour perfusion analysis. (**d**) Paraaxial CT image from the dVPCT stack perpendicular to the rectum lumen, with the corresponding representative semicircular tumour area at 3 to 9 o’clock suitable for tumour perfusion analysis.

**Figure 3:**
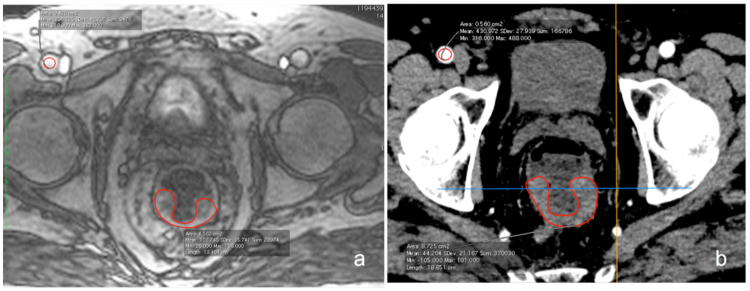
Example of corresponding rectal cancer tumour areas and positioning of ROIs for the assessment of arterial input function (AIF) for perfusion: (**a**) Example of a manually drawn ROI for assessment of AIF over the right superficial femoral artery and a manually drawn ROI for perfusion analysis over a representative slice of tumour area on MR perfusion images. (**b**) Example of the manually drawn ROI for assessment of the arterial input function (AIF) over the right superficial femoral artery in analogy to (**a**) and a manually drawn ROI for perfusion analysis in the identical representative slice of tumour area on dVPCT images corresponding to (**a**).

MR: Perfusion quantification was performed as follows: a region-of-interest (ROI) was carefully manually drawn to delineate the tumour in a representative slice within the maximum area of circumferential tumour burden (see Figs 2 and 3), as well within normal rectal wall distant from the tumour. To estimate the arterial input function (AIF), another ROI was drawn in one of the iliac arteries (common or external) or femoral superficial artery visible (see Fig. 3) depending on the specific tumour position within the rectum using the Osirix DICOM viewer (Pixmeo, Geneva, Switzerland). Perfusion parameters were calculated employing the fast deconvolution algorithm using an in-house developed established perfusion plugin tool (UMMPerfusion)^23^. Plasma flow (PF) [mL/100 mL/min], plasma volume (PV) [mL/100 mL] and mean transit time (MTT) [s] were estimated in tumour tissue and normal rectal wall.

CT: The noise-reduced and motion-corrected dVPCT data stack was evaluated as follows: ROIs delineating the tumour tissue and normal rectal wall as well as the AIF were drawn in the identical regions and the identical vessel (see Fig. 3) using dedicated software (Siemens syngo.via CT Body Perfusion VA30, Siemens Healthineers, Erlangen, Germany). A variant of the deconvolution algorithm was applied to calculate dVPCT values. Blood flow (BF) [mL/100 mL/min], blood volume (BV) [mL/100 mL], MTT [s] and Permeability (PERM) [mL/100 mL/min] were estimated in tumour tissue and normal rectal wall.

Deconvolution approaches in general assume that the tissue time concentration curve $C(t)$ can mathematically be described as convolution of an AIF ${C}_{A}(t)$ with an unknown impulse residue/response function (*IRF*)^20^. In this equation, the *IRF* defines the probability that a molecule of the agent entering the tissue voxel at time-point *t*′ is still there at time-point $t$.

1 $$C(t)=\,{\int }_{{\rm{\Delta }}t}^{t}{C}_{A}(t^{\prime} -{\rm{\Delta }}t)\times IRF\,(t-t^{\prime} )\times dt^{\prime} $$

In the CT software used in this study a variant of the deconvolution algorithm, namely the AATH model (short for adiabatic approximation to the tissue homogeneity model) including determination of the arterial arrival delay $\bigtriangleup t$ in equation 1 was applied^20^. In this form it has 6 free parameters: baseline attenuation and arrival delay $\bigtriangleup t$, blood flow F, mean transit time MTT, flow extraction product FE and a decay parameter containing the extravascular distribution volume^20^. In the equation of the impulse residue function IRF of the AATH model, the blood volume BV is calculated from the central volume principle as BV = F × MTT. PS - or PERM as it is called in our study - (permeability surface area product) is calculated from E (extraction fraction) = FE/F using the Renkin-Crone equation)^20^. For MR perfusion analysis on the other hand, a fast (model-free) deconvolution algorithm was applied in our study:

2 $$f=\,{\rm{\max }}\,[{C}_{t}(t)\,{\otimes }^{-1}\,{C}_{a}(t)]$$

here, ${C}_{t}(t)$ and ${C}_{a}(t)$ are the contrast material concentrations as the function of time in a region-of-interest (ROI) in the tissue and inside the artery feeding the region of interest (arterial input function), respectively^24^. The perfusion $f$ is the maximum value of the tissue impulse response function, i.e. the deconvolution (⊗^−1^) of the two concentration functions^24^. Eq. 2 is solved by singular value decomposition (SVD) using a regularization of 0.15 times the maximal singular value^24^.

The reading was performed by one radiologist (T.H.) with 7 years of experience in abdominal MRI and CT. The complete analysis was performed two times (M1 and M2 with >2 weeks between the 2 time points) to assess intra-reader variability.

### Statistical analysis

Statistical analysis was performed using dedicated software (SAS, Institute Inc. Software, version 9.4, 2012 (Tests); Prism Version 7.03 for Windows, GraphPad Software, La Jolla, California, USA, www.graphpad.com; JMP, Version 13.0.0. SAS Institute Inc.; Cary, NC 1989–2007). For the descriptive analysis mean, standard deviation, median, range, skewness and kurtosis of distribution have been assessed for the continuous variables. Variability of measurements is described by the coefficient of variation (CoV) consisting of the percentage of the standard deviation from the means. Differences between duplicate measurements of the same reader are described by the intra-class correlation coefficient (ICC) and its 95% confidence interval (CI) and results of Bland-Altman analysis. Here, beside the p-value, the arithmetic mean difference of M1 and M2 (95% CI) is given to further describe intra-reader variability of the data. Given the results of d’Agostino-Pearson-tests for the variables, non-parametric testing was further performed: the Wilcoxon sign rank test (for paired data) was used to investigate differences between tumour tissue and non-diseased rectal wall in dVPCT and perfusion MRI parameters. The level of significance was set to p = 0.05.

The datasets generated during and/or analysed during the current study are available from the corresponding author on reasonable request.

## Results

In Table 2 absolute values of the 4 dVPCT parameters and the 3 MR-perfusion parameters, respectively, are listed for measurements M1 and M2 in tumour tissue and normal rectal wall.

**Table 2:** Variability of perfusion measurements in tumour tissue and normal rectal wall with MR-Perfusion vs. dVPCT.

Perfusion Parameter	MR-Perfusion M1		MR-Perfusion M2		dVPCT M1		dVPCT M2	
	Tumour	Rectal wall	Tumour	Rectal wall	Tumour	Rectal wall	Tumour	Rectal wall
Plasma flow/Blood flow [mL/100 mL/min]	43.9 ± 23.4 (±53%)	33.9 ± 16.3 (±48%)	51.7 ± 24.2 (±47%)	34.8 ± 16.5 (±47%)	78.4 ± 23.0 (±29%)	28.3 ± 10.4 (±37%)	80.2 ± 23.5 (±29%)	27.9 ± 9 (±32%)
Plasma volume/Blood volume [mL/100 mL]	40.9 ± 25.8 (±63%)	28.7 ± 17.8 (±62%)	48.0 ± 35.7 (±74%)	27.6 ± 19.2 (±70%)	6.0 ± 1.7 (±28%)	2.2 ± 0.7 (±32%)	6.1 ± 1.7 (±28%)	2.2 ± 0.7 (±32%)
Mean transit time [s]	60.1 ± 24.6 (±41%)	50.1 ± 18.2 (±36%)	56.7 ± 24.3 (±43%)	44.0 ± 17.2 (±39%)	5.4 ± 1.4 (±26%)	5.3 ± 0.8 (±15%)	5.3 ± 1.2 (±23%)	5.5 ± 0.8 (±15%)
Permeability [mL/100 mL/min]					24.1 ± 7.6 (±32%)	8.3 ± 3.2 (±39%)	25.0 ± 6.9 (±28%)	9.0 ± 3.5 (±39%)

Legend: Data means ± standard deviation (± coefficient of variation (CoV).

Variability of MR measurements was generally higher than of dVPCT measurements (see Table 2) with an average coefficient of variation of PF/BF: 50% vs. 29% for tumour tissue and 48% vs. 35% for normal rectal wall, respectively; of PV/BV: 69% vs. 28% for tumour tissue and 66% vs. 32% for normal rectal wall, respectively, and MTT: 42% vs. 25% for tumour tissue and 38% vs. 15% for normal rectal wall, respectively.

Intra-reader variability analysis revealed no systematic differences between duplicate dVPCT measurements in tumour tissue (p values between 0.45–0.87) with ICC between 0.68 and 0.89 or normal rectal wall (p values between 0.30 and 0.96) with ICC between 0.50 and 0.68. In MRI, same was true for PF, PV and MTT in tumour tissue (p values between 0.13 and 0.34) with ICC between 0.46 and 0.92, whilst for normal rectal wall p values were solely non-significant for PF and PV (0.82 and 0.23), but not for MTT with ICC between 0.64 and 0.93, see Tables 3 and 4. Mean relative difference between M1 and M2 was much lower in dVPCT-derived BF, BV and MTT for tumour tissue compared to MR-Perfusion-derived PF, PV and MTT (PF −16% vs. BF −2%; PV −9% vs. BV −2% and MTT 7% vs. 0.4%), and same was true for the three comparable measurements in normal rectal wall: PF −1% vs. BF 0.4%; PV 10% vs. BV −3% and MTT 14% vs. −4% (see also Table 4).

**Table 3:** Assessment of Intra-reader variability of MR-Perfusion and dVPCT – Intraclass correlation coefficient analysis.

Perfusion Parameters	MR-perfusion				Perfusion Parameters	dVPCT			
	M1 vs. M2					M1 vs. M2			
	Tumour		Rectal wall			Tumour		Rectal wall	
	Intraclass correlation coefficient^a^	95%-CI	Intraclass correlation coefficient^a^	95%-CI		Intraclass correlation coefficient^a^	95%-CI	Intraclass correlation coefficient^a^	95%-CI
Plasma flow [mL/100 mL/min]	0.4638	0.04–0.75	0.9333	0.84–0.97	Blood flow [mL/100 mL/min]	0.7691	0.50–0.90	0.6015	0.23–0.82
Plasma volume/ [mL/100 mL]	0.5773	0.19–0.81	0.8223	0.60–0.93	Blood volume [mL/100 mL]	0.8912	0.75–0.96	0.6790	0.35–0.86
Mean transit time [s]	0.9240	0.8–0.97	0.6421	0.29–0.84	Mean transit time [s]	0.8699	0.70–0.95	0.4963	0.08–0.76
					Permeability [mL/100 mL/min]	0.6847	0.36–0.86	0.6165	0.25–0.83

^a^Estimates the reliability of single ratings. CI = confidence interval.

**Table 4:** Assessment of Intra-reader variability of MR-Perfusion and dVPCT – Bland-Altman analysis.

Perfusion Parameters	MR-perfusion				Perfusion Parameters	dVPCT			
	M1 vs. M2					M1 vs. M2			
	Tumour		Rectal wall			Tumour		Rectal wall	
	p-value (H_0_: Mean = 0)	Arithmetic mean difference (95%-CI) [%]	p-value (H_0_: Mean = 0)	Arithmetic mean difference (95%-CI) [%]		p-value (H_0_: Mean = 0)	Arithmetic mean difference (95%-CI) [%]	p-value (H_0_: Mean = 0)	Arithmetic mean difference (95%-CI) [%]
Plasma flow [mL/100 mL/min]	0.1292	−15.89 (−36.85–5.08)	0.8233	−1.01 (−10.30–8.29)	Blood flow [mL/100 mL/min]	0.6439	−2.09 (−11.41–7.23)	0.9599	0.38 (−15.31–16.07)
Plasma volume/ [mL/100 mL]	0.3435	−9.12 (−28.73–10.52)	0.2275	9.74 (−6.61–26.09)	Blood volume [mL/100 mL]	0.4788	−2.02 (−7.86–3.83)	0.6616	−3.35 (−19.10–12.41)
Mean transit time [s]	0.1917	7.29 (−3.98–18.56)	0.0409*	14.33 (0.65–28.01)	Mean transit time [s]	0.8732	0.39 (−4.65–5.43)	0.3034	−3.45 (−10.27–3.38)
					Permeability [mL/100 mL/min]	0.4476	−4.38 (−16.21–7.44)	0.3721	−7.49 (−24.63–9.66)

Arithmetic mean differences are presented as % from the mean of M1 and M2. CI = confidence interval. ^*^ indicates statistical significance.

Significantly different values for tumour tissue and normal rectal wall were found for three of the four dVPCT parameters, with M1 tumour vs. M1 normal rectal wall a BF of 78.4 vs. 28.3 mL/100 mL/min; BV of 6.0 vs. 2.2 mL/100 mL and PERM of 24.1 vs. 8.3 ml/100 mL/min, with all three p values < 0.0001, and M2 tumour vs. M2 normal rectal wall showing for the same three parameters all significant p values < 0.0001 as well; see Table 1 for MTT and all the detailed absolute M2 tumour and M2 normal rectal wall values. Regarding MRI, PF and PV showed significantly different values for tumour tissue and normal rectal wall in both M1 and M2 comparisons, while equivocal results were found for M1 and M2 again regarding MTT, see Table 5.

**Table 5:** Tumour vs. normal rectal wall in MR-perfusion and dVPCT.

Perfusion parameters	MR-perfusion Tumour vs. rectal wall				dVPCT Tumour vs. rectal wall			
	M 1		M2		M 1		M 2	
	p-value	Est. Median Diff (95%-CI)	p-value	Est. Median Diff (95%-CI)	p-value	Est. Median Diff (95%-CI)	p-value	Est. Median Diff (95%-CI)
Plasma flow/ Blood flow [mL/100 mL/min]	0.0400*	7.895 (−2.31–15.79)	0.0107*	11.1 (−0.3–26.3)	<0.0001*	45.8 (30.57–60.73)	<0.0001*	53.73 (32.56–65.83)
Plasma volume/ Blood volume [mL/100 mL]	0.0020*	10.50 (8.615–19.32)	0.0028*	13.65 (3.9–28.4)	<0.0001*	3.525 (2.93–3.95)	<0.0001*	3.795 (2.54–4.91)
Mean transit time [s]	0.0696	11.12 (−0.27–22.31)	0.0266*	7.75 (6.0–23.7)	0.9273	−0.13 (−0.63–0.53)	0.3884	−0.415 (−0.93–0.3)
Permeability [mL/100 mL/min]					<0.0001*	14.09 (10.78–18.25)	<0.0001*	17.33 (9.56–21.28)

CI = confidence interval. ^*^ indicates statistical significance.

Regarding radiation dose, 19/20 patients were scanned with a combination of 80 kV/220 mAs tube voltage/tube current. One patient was scanned with the combination of 100 kV/100 mAs tube voltage/tube current.

Mean dose-length product of the dVPCT study sequence performed over the z-axis of the tumour was 1569 ± 367 mGycm (min. 1011 to max. 2389 mGycm), corresponding to an effective dose of 20.4 ± 4.8 mSv, if a conversion factor of 0.013 for the pelvis according to ICRP Publication 103 is used^25^. Mean dose-length product of the total CT examination including the dVPCT study sequence was 2243 ± 451 mGycm (min. 1489 to max. 3476 mGycm), corresponding to an effective dose of 29.2 ± 5.9 mSv, if a conversion factor of 0.013 for the perfusion CT in the pelvic region and a conversion factor of 0.015 for the scans of the chest and abdomen are used according to ICRP Publication 103.

## Discussion

We found that 3^rd^ generation noise-reduced and motion-corrected dVPCT parameters assessed in patients with rectal cancer have a generally lower intra-individual variability than MR-perfusion parameters, which is true in tumour tissue as well as in normal rectal wall. What is more, dVPCT measurements were found to be reproducible within narrow variation ranges both in rectal cancer tumour tissue and normal rectal wall in the intra-reader variability analysis. We believe that the noise-reduction and motion-correction post-processing algorithms applied before CT perfusion analysis in this study might have added to the observed lower data variability in the first place. If similar post-processing algorithms are going to be established for MR perfusion analysis, the cards will supposedly be reshuffled again.

Our third finding is that dVPCT can differentiate tumour tissue and normal rectal wall by means of BF, BV and PERM measurements. Similar findings were published by Bellomi *et al*. who found that BF, BV, and permeability-surface area product (PS) were significantly higher in rectal cancer than in normal rectal wall^26^. Bellomi *et al*. were using a 16-section multi-detector CT scanner. MTT seems to be rather unhelpful in distinguishing rectal cancer tumour tissue from normal rectal wall. This seems to be true for both dVPCT and MR-perfusion on the basis of our results. Albeit we used similar tracer kinetics models for the calculation of perfusion values in CT and MRI, the deconvolution algorithm-derived (AATH model) absolute dVPCT measurements cannot be directly compared with the fast deconvolution algorithm-derived absolute MR-perfusion measurements as explained in great detail above referring to the publications by Klotz *et al*.^20^ and Gaa *et al*.^24^. As the approach of the MR perfusion analysis, the fast (model-free) deconvolution algorithm, does not describe two compartments, several perfusion parameters as e.g. permeability surface area product cannot be determined. Therefore, this tracer kinetics model might assumedly not be as interesting for anti-vascular drugs treatment monitoring and response assessment as e.g. two compartment models, where the assumption is made that the tracer in the tissue can distribute in two separate compartments, namely the plasma space and the extracellular extravascular space/interstitial space^24^. What is more, irrespective of the tracer kinetics model being applied after data acquisition, different contrast bolus application protocols and different lengths as well as differing time-points of data acquisition of the perfusion sequences (also resulting in a different temporal resolution at different time-points) are used in CT compared to MRI. Albeit one study by Kierkels *et al*.^27^ that compared absolute values of CT perfusion performed on a 40-slice CT-positron emission tomography system and MR perfusion performed on a 3 T scanner system and found that the values of the transfer constant K(trans) did correlate, while fractional extravascular-extracellular space v(e), and fractional plasma volume v(p) did not, one can generally say that CT and MR rectal cancer perfusion analyses cannot alternatingly be used for assessing therapeutic response in patients. Consequently, it is of great interest to define the modality with the best capability to differentiate tumour tissue, with low variability of the data and good reproducibility.

One disadvantage of CT perfusion for to date is the necessity of an extra i.v. contrast bolus for the perfusion sequence. However, with technical advantage and broader use of scanners where low kV settings at sufficiently high tube current power are available, it would also be possible to add a larger range venous scan (thorax and abdomen) for staging purposes immediately after the perfusion study without the need for a second contrast injection. Although, the total amount of iodine applied to the patient would be lower, future studies will probably show that this can be fully compensated for by the increased iodine contrast at low kV^20^.

The biggest disadvantage of dVPCT is the amount of radiation dose which is still applied despite the noise-reduction and motion-correction post-processing algorithms applied before CT perfusion analysis that have led to the possibility to a-priori reduce the dose of the perfusion scan: even by using the shortest possible z-axis coverage for the dVPCT sequence and by using a 3^rd^ generation dual-source CT scanner system, radiation dose of the total CT examination including the perfusion sequence amounted to an average DLP of 2243 mGycm. Albeit not exceeding reference radiation dose levels of the Federal Office for Radiation Protection in Germany for a standard CT examination of the chest and a biphasic scan of the abdomen which at the time of data acquisition was estimated at a DLP of 2200 mGycm^22^, still, in our study protocol, the dVPCT sequence accounted for at average 1569 mGycm or 70% of the total dose.

## Limitations

This analysis was performed with a small patient cohort and further research is needed to verify findings. Multiple publications investigated the diagnostic and prognostic value of CT perfusion with regard to tumour histology, presence of distant metastasis, therapeutic response to chemo-radiation therapy as well as overall survival^16,28–30^. We did not correlate perfusion parameters with histo-pathological findings, surgical data or outcome data. Furthermore, this analysis focused on absolute perfusion parameter values. However, recently performed research suggests semi-quantitative analysis of pharmacokinetic curves in MR-perfusion as valuable parameter for prediction of tumour aggressiveness and response in rectal cancer^8,31^. Yet, semi-quantitative perfusion analysis was out of the scope of this paper. A crucial bias that all perfusion examinations in CT and MRI are facing is the calculation of the AIF. The approach of this paper was to keep CT ROI for AIF constant to the ROI chosen for MR-perfusion analysis, namely common or external iliac arteries or superficial femoral artery. However, as the blood supply of the rectum is complex, and iliac arteries are often prone to present with plaques and stenosis in patients with atherosclerosis, the authors know, that this method can only be a rough approximation of the real AIF. There are multiple approaches to minimize the bias caused by AIF calculation, e.g. by calculating semi-automated and threshold-based a mean AIF from all vessels visible in the perfusion sequences, yet, this approach was out of the scope of this publication.

What was discussed already in great detail above is that the deconvolution algorithm is only one possibility of many to estimate perfusion. It is known, that perfusion values largely differ between different tracer kinetics models, e.g. if compartment models are applied^24,32^.

Perfusion analysis results might also vary even within the same imaging modality depending on temporal resolution being dependent on z-axis coverage and on cycle protocols if shuttle mode CT perfusion is applied as well as on acquisition duration^20,33^. Goh *et al*. found that increasing the temporal interval from 1 to 4 seconds leads to overestimation of tumour blood flow and underestimation of blood transit in distributed parameter analysis^18^. Therefore, acquisition interval should be shorter than 3 seconds, which was the case with 1.5 s cycle time during arterial inflow phase in our CT studies. Yet, with regard to radiation dose, cycle time was increased up to 9 s towards the end of the 64 s of the perfusion scan. Finally, it is known that perfusion analysis values might even vary due to different versions of the same perfusion software used^34^.

We did not include inter-reader variability analysis in this paper, but previous publications have investigated this topic for CT^35^ and MRI, respectively^7^.

We did not perform detailed evaluation of subjective or objective image quality.

CT scan protocol was designed to adhere to national diagnostic reference values by the German Federal Office for Radiation Protection^22^ for standard CT chest and abdomen examinations valid at the time-point of patient enrolment. The possibility to include with new CT scanner systems dose-intensive perfusion sequences in CT staging protocols without exceeding dose reference values is very encouraging given accuracy and validity of the perfusion analysis. Given the fact, that there are even attempts of the CT community to overtake T staging^36^ which is to date clearly regarded as the holy grail of MR in rectal cancer imaging, the idea of a one-stop-shop CT approach in rectal cancer patients quickly comes to one’s mind. However, as dose recommendations for standard CT examinations are constantly updated and national CT dose reference values have been only recently lowered dramatically^22,37^, further optimization of perfusion CT protocols taking e.g. acquisition duration for dose reduction into account is mandatory to reach this goal in the future^20,38^.

## Conclusions

Our data show that 3^rd^ generation dual-source deconvolution model- derived dVPCT measurements have a lower intra-individual variability than MR-perfusion measurements in rectal cancer patients and are also highly reproducible between the same reader within narrow variation ranges in rectal cancer tumour tissue as well as in normal rectal wall. dVPCT can differentiate tumour tissue and normal rectal wall by means of BF, BV and PERM measurements. With a 3^rd^ generation dual-source CT scanner system, dVPCT could be included into a chest and abdomen CT staging examination in our study without exceeding national diagnostic reference value recommendations.

## Electronic supplementary material

Video legends

Video 1

Video 2
