# Supplementary material for: Variability and Reproducibility of 3rd-generation dual-source dynamic volume perfusion CT Parameters in Comparison to MR-perfusion Parameters in Rectal Cancer
Source: Sci Rep. 2018 May 2;8:6868. doi: 10.1038/s41598-018-25307-w (PMC5932032; doi:10.1038/s41598-018-25307-w)
Supplement: Supplementary file 1 — Video legends [file 41598_2018_25307_MOESM1_ESM.docx]

**“Variability and Reproducibility of 3^rd^-generation dual-source dynamic volume perfusion CT Parameters in Comparison to MR-perfusion Parameters in Rectal Cancer”**

*Sonja Sudarski, MD^1^; Thomas Henzler, MD^1^, Teresa Floss, BS^1^, Tanja Gaa, M.Sc.^2^, Mathias Meyer, MD^1^, Holger Haubenreisser, MD^1^, Stefan O. Schoenberg, MD^1^; Ulrike I. Attenberger, MD^1^

1. Institute of Clinical Radiology and Nuclear Medicine, University Medical Centre Mannheim, Medical Faculty Mannheim, Heidelberg University, Germany
2. Computer Assisted Clinical Medicine, Medical Faculty Mannheim, Heidelberg University, Mannheim

Corresponding author:

*Sonja Sudarski, M.D.

Institute for Clinical Radiology and Nuclear Medicine, University Medical Centre Mannheim,

Medical Faculty Mannheim - Heidelberg University

Theodor-Kutzer-Ufer 1-3, D-68167 Mannheim, Germany

Tel.: (+49) (+621) 383 - 2067

Fax: (+49) (+621) 383 - 1910

E-Mail: [sonja.sudarski@medma.uni-heidelberg.de](mailto:sonja.sudarski@medma.uni-heidelberg.de)

**Video legends:**

Video 1:

Example of a 66 year-old patient with newly-diagnosed rectal cancer prior to neoadjuvant chemotherapy and surgery with corresponding perfusion sequences of his semicircular carcinoma at 3 to 9 o’clock in the lithotomy position. Axial view of MR Perfusion.

Video 2:

Example of a 66 year-old patient with newly-diagnosed rectal cancer prior to neoadjuvant chemotherapy and surgery with corresponding perfusion sequences of his semicircular carcinoma at 3 to 9 o’clock in the lithotomy position. Axial view of dVPCT at 80 kVp, 220 mAs.
